# The Role of the Discharge Planning Team on the Length of Hospital Stay and Readmission in Patients with Neurological Conditions: A Single-Center Retrospective Study

**DOI:** 10.3390/healthcare13020143

**Published:** 2025-01-14

**Authors:** Anmar Fatani, Sarah Alzebaidi, Himyan Kamel Alghaythee, Suzan Alharbi, Mohammed Hisham Bogari, Hassan K. Salamatullah, Saeed Alghamdi, Seraj Makkawi

**Affiliations:** 1 Department of Neurology, Ministry of The National Guard Health Affairs, Jeddah 22384, Saudi Arabia; 2 King Abdullah International Medical Research Center, Jeddah 22384, Saudi Arabia; 3 Department of Ophthalmology, Jeddah Eye Hospital, Jeddah 23454, Saudi Arabia; 4 College of Medicine, King Saud Bin Abdulaziz University for Health Science, Jeddah 22384, Saudi Arabia; 5 Neuroscience Department, King Faisal Specialist Hospital and Research Center, Jeddah 21556, Saudi Arabia

**Keywords:** neurology, discharge planning, length of stay, readmission, hospitalization, mortality, Saudi Arabia

## Abstract

**Background:** Emerging evidence highlights the critical role of discharge planning teams in enhancing patient care. However, there is lack of data regarding how the implementation of a discharge planning system influences the length of stay (LOS) in hospital and readmission rates among patients with neurological diseases. We conducted a retrospective analysis to examine the effects of discharge planning application on the LOS and readmission rates for patients admitted under the neurology service in Saudi Arabia. **Methods:** This is a retrospective study conducted at King Abdulaziz Medical City—Western region of Saudi Arabia. We included all patients admitted and discharged under the neurology service between January 2018 and December 2019. The included patients who were divided into the 2018 group (not exposed to discharge planner) and the 2019 group (exposed to discharge planner). The primary endpoints were the LOS and readmission rates. **Results:** The study included 856 patients (436 allocated to the 2019 group and 420 allocated to the 2018 group). There was no difference between the two groups in the LOS after adjusting confounding factors (β = −0.58, 95% CI [−2.79, 1.61], *p* = 0.60). However, the 2019 group were more likely to be discharged within three days compared to the 2018 group (41% vs. 26%, *p* < 0.005). Our analysis showed that patients in the 2019 group were less likely to be readmitted compared to the 2018 group (adjusted odds ratio = 0.70, 95% CI [0.49–0.99], *p* = 0.0442). **Conclusions:** Implementation of a discharge planning team was associated with higher early discharge rates and lower hospital readmissions, suggesting potential benefits for healthcare resource utilization in neurological services.

## 1. Introduction

Neurological diseases represent a major cause of long-term disability, leading to significant physical and psychosocial impacts for both patients and their families [1]. The Global Burden of Disease Study highlights this issue, revealing that neurological disorders were responsible for approximately 276 million disability-adjusted life years and 9 million deaths worldwide in 2016 [2]. Further illustrating the severity of these conditions, a study focused on patients recovering from an acute stroke shows that these individuals often require extended hospital stays due to the need for intensive rehabilitation and management of complications [3]. Importantly, evidence shows that a multidisciplinary approach, including a discharge planner, can lead to earlier discharges, improved functional independence, and fewer patients requiring long-term institutional care [4,5].

The discharge planner is essential in developing personalized discharge plans for patients from their first day of admission, enhancing communication among hospital teams and coordinating with external transfer services as needed [6,7]. A randomized clinical trial showed that discharge planners effectively reduce hospitalization durations and improve patient satisfaction for chronic obstructive pulmonary disease [8]. While healthcare professionals traditionally focused on medical care, non-medical factors, such as unstable housing and lack of community support, significantly hinder timely discharges [9]. Inadequate assessments, delays in transportation arrangements, and necessary transfers across departments can prolong stays, increase bed occupancy, and raise costs [9,10]. The literature presents conflicting evidence regarding the effectiveness of discharge planning interventions. While some studies demonstrate clear benefits, others show minimal or no impact on key outcomes. Systematic reviews indicate that multidisciplinary stroke units with integrated discharge planning can lower mortality rates, shorten hospital stays, and reduce dependency levels, proving to be cost-effective [11,12]. Braet et al.’s systematic review found that discharge planning interventions reduced hospital readmissions by 22% [13], whereas a meta-analysis reported by Mistiaen et al. showed no significant effect on readmission rates in certain patient populations [14]. Similarly, a Cochrane review conducted by Gonçalves-Bradley et al. reported a small reduction in hospital stays (mean difference −0.73 days, 95% CI −1.33 to −0.12) [7], while a more recent systematic review conducted by Hunt-O’Conner and colleagues revealed inconclusive findings in terms of the length of stay (LOS) in hospital and readmission rates after application of a discharge planning team across different healthcare settings [15]. The variability in outcomes may be attributed to differences in discharge planning models, implementation strategies, healthcare systems, and patient populations. For instance, Naylor et al. demonstrated that comprehensive discharge planning with home follow-up significantly reduced readmissions in older adults with heart failure [16], while Shepperd et al. found no clear impact on health outcomes in a mixed patient population [17]. These contradictory findings highlight the need for more targeted research examining the effectiveness of discharge planning in specific patient populations and healthcare contexts.

Most of these studies were mainly performed in the Western countries, leaving a significant gap in understanding the role of discharge planners in Saudi Arabia, especially in patients with neurological conditions. Our study aims to compare patients with neurological conditions who engaged with a discharge planning team to those who did not, focusing on the LOS and readmission rates.

## 2. Materials and Methods

### 2.1. Ethical Approval

This study was conducted in accordance with the Declaration of Helsinki and approved by the Institutional Review Board (IRB) of the King Abdullah International Medical Research Center (KAIMRC) under study number RJ20/237/J on 7 February 2021.

### 2.2. Study Design and Patient Selection

Our study adhered to Strengthening the Reporting of Observational Studies in Epidemiology (STROBE) reporting guidelines [18]. This study was a retrospective study conducted at the National Guard Health Affairs (NGHA), a tertiary care center located within King Abdullah Medical City in Jeddah (KAMC-J). The research period spanned from January 2018 to December 2019. Notably, prior to April 2019, the discharge planning team had not yet been established at our institution. The patient cohort was divided into two distinct groups based on their admission dates and exposure to the discharge planning team. The first group comprised patients admitted under the neurology service from January 2018 to March 2019, who were not exposed to the discharge planning team (2018 group). Conversely, the second group included patients admitted under the same service from April 2019 to December 2019, who were exposed to the discharge planning team (2019 group). The sampling method employed for this study was a non-random convenience sampling technique. To fulfill the inclusion criteria, patients who were admitted and discharged under the neurology service between January 2018 and December 2019 were included. We excluded patients who were admitted and discharged from day-case units, and those receiving care at other hospitals or departments.

At our institution, the discharge planning team operates under the umbrella of bed management, reporting to the executive medical services to ensure efficient action and timely execution. This team consists of administrative case managers who facilitate the implementation of orders prescribed by the primary medical team. Their responsibilities are dual in nature. Firstly, they enhance communication between the medical team and other hospital departments, such as radiology services, ensuring that patients requiring various imaging procedures such as brain magnetic resonance imaging (MRI) scans, computed tomography (CT) scans, and CT angiograms receive them promptly. Additionally, they expedite the completion of physician orders, including Holter monitoring, echocardiograms, and electroencephalograms (EEGs), ensuring their swift execution. Secondly, the discharge planning team organizes post-discharge follow-up appointments and coordinates services and orders based on the medical team’s assessment of the patient’s needs. For patients requiring additional support, they arrange appointments with physiotherapy, occupational therapy, and home healthcare services. They also collaborate with social workers to ensure patients’ homes are appropriately modified and equipped to accommodate disabilities or special needs. This administrative team plays a critical role as an intermediary between the medical team, support services, and post-discharge care providers, optimizing the transition from hospital to home care.

### 2.3. Data Collection

A pre-established Excel sheet was utilized to obtain information from the institution’s medical records. The data sheet was divided into four sections. The data sheet included information regarding demographics (age, gender, body mass index [BMI]), past medical history (diabetes, hypertension, dyslipidemia, stroke, transient ischemic attack [TIA], atrial fibrillation, heart failure, chronic kidney disease, coronary artery disease, liver cirrhosis, hemodialysis, peripheral vascular disease), chief complaint of the current admission (reason for admission, date of admission, LOS), hospital course (immediate intensive care unit [ICU] admission, ICU admission, pneumonia, urinary tract infection, myocardial infarction [MI], pulmonary embolism, dysphagia, intravenous [IV] antibiotic requirement), and Barthel index.

### 2.4. Outcomes

The primary endpoints were the LOS, defined as the duration from the first day of admission to the day of discharge, and readmission rates after discharge. Secondary outcomes included mortality during the same admission, and overall mortality following discharge.

### 2.5. Statistical Analysis

Statistical analyses were conducted using J Macintosh Project pro (JMP version 17.2.0). The distribution of length of stay (LOS) data were assessed using the Kolmogorov–Smirnov and Shapiro–Wilk tests, which revealed non-normal distribution; therefore, LOS data were presented as the median with the interquartile range (IQR), and Mann–Whitney U tests were employed for between-group comparisons. Categorical variables were reported as frequencies and percentages and compared using the chi-square test. A multiple linear regression analysis was performed to examine the association between discharge planning team implementation and the LOS while controlling for potential confounders. The model included demographic variables (age, gender, BMI), comorbidities (diabetes, hypertension, dyslipidemia, stroke history, TIA, atrial fibrillation, heart failure, chronic kidney disease, coronary artery disease, liver cirrhosis, hemodialysis, peripheral vascular disease), hospital course variables (immediate ICU admission, ICU admission, pneumonia, urinary tract infection, myocardial infarction, deep vein thrombosis, pulmonary embolism, dysphagia, IV antibiotic requirement), and functional status (Barthel index). The results were presented as the β coefficient and 95% confidence interval (CI). To investigate the interaction between the significant independent variables in the model with the main independent variable (discharge planning team exposure), an interaction analysis was undertaken. The interaction terms were created based on which independent variable was significant and clinically correlated in the model. For categorical analyses of the LOS, patients were stratified into groups based on the duration of their stay (1–3 days, 4–7 days, 8–14 days, 15–30 days, and >30 days), and distributions were compared between the 2018 and 2019 cohorts using chi-square tests. Subgroup analyses on the LOS were performed for different neurological conditions using the same statistical approaches. For the binary outcomes (readmission, same-admission mortality, and overall mortality), a logistic regression model was constructed using the same independent variables to calculate adjusted odds ratios (ORs) with a 95% CI. Readmission periods were categorized into three time intervals after discharge: within one month, between 2 and 12 months, and beyond 12 months. The distribution of readmissions across these time periods was analyzed using chi-square tests to compare patterns between the 2018 and 2019 cohorts. All statistical tests were two-tailed, with statistical significance set at *p* < 0.05.

## 3. Results

### 3.1. Baseline Characteristics

The study included a total of 856 patients, with 420 (49%) assigned to the 2018 group and 436 (51%) assigned to the 2019 group. The study population consisted of 485 (57%) males and 371 (43%) females. The median age of the participants was 63 years in the 2018 group, while it was 62 years of age in the 2019 group. The median Barthel index was equal between the two groups. Further baseline characteristics are presented in Table 1.

Among the patients admitted to the neurology department, 304 (36%) were diagnosed with an ischemic stroke, 50 (6%) with a hemorrhagic stroke, 51 (6%) with a transient ischemic attack, 90 (11%) with seizures, 53 (6%) with a multiple sclerosis relapse, 16 (2%) with a myasthenia gravis crisis, and 36 (4%) with meningitis.

During the hospital admission, patients in the 2018 group were more likely to have urinary tract infection and deep vein thrombosis, and were more likely to be admitted to the ICU compared to patients in the 2019 group (Table S1).

### 3.2. Length of Hospital Stay

**Figure 1:**
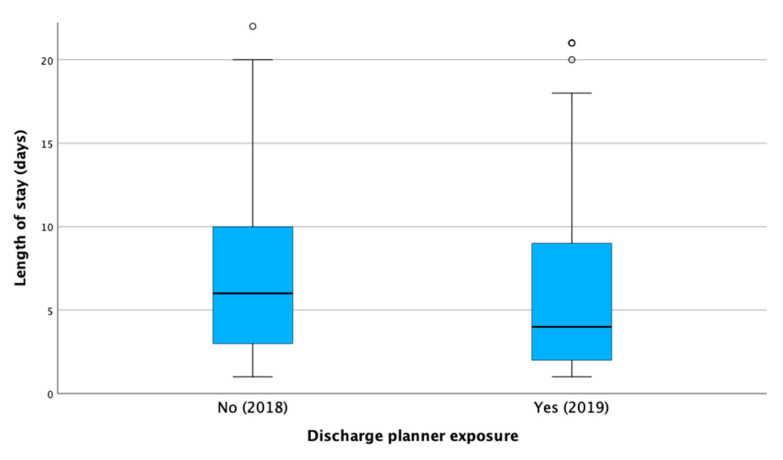
The difference in length of stay in hospital between 2018 group and 2019 group.

The results showed that patients admitted in 2018 had a significantly higher mean rank of LOS (467.78) compared to those admitted in 2019 (390.66), with a *p*-value < 0.001. The descriptive statistics further support this finding, with the median LOS decreasing from 6 days (3–10) for patients in the 2018 group to 4 days (2–9) for those in the 2019 group (Figure 1).

The multiple linear regression analysis was conducted to examine the association between the implementation of a discharge planning team and LOS while controlling for various patient characteristics and clinical factors. After adjusting for the other variables, the presence of a discharge planning team was not significantly associated with the LOS (β = −0.58, 95% CI [−2.78, 1.61], *p* = 0.60). However, several other factors were found to be significantly associated with the LOS. Patients admitted to the ICU (*p* < 0.0001) and those requiring intravenous antibiotics (*p* < 0.0001) had significantly longer hospital stays. The presence of certain comorbidities also contributed to an increased LOS, including liver cirrhosis (*p* = 0.03), and dysphagia (*p* = 0.02). In terms of functional status, each increase in the Barthel index was associated with a shorter LOS (*p* = 0.0045) (Table S2).

An interaction analysis was performed between the discharge planning team exposure and the following variables: liver cirrhosis, immediate ICU admission, ICU admission, dysphagia, IV antibiotic requirement, and the Barthel index. There was significant interaction between discharge planning team exposure and liver cirrhosis (β = 14.39, 95% CI [1.87, 26.9], *p* = 0.0243), immediate ICU admission (β = 5.61, 95% CI [0.44, 10.77] *p* = 0.0335), and IV antibiotic requirement (β = −3.35, 95% CI [−6.49, −0.21] *p* = 0.0363) (Table S3).

**Figure 2:**
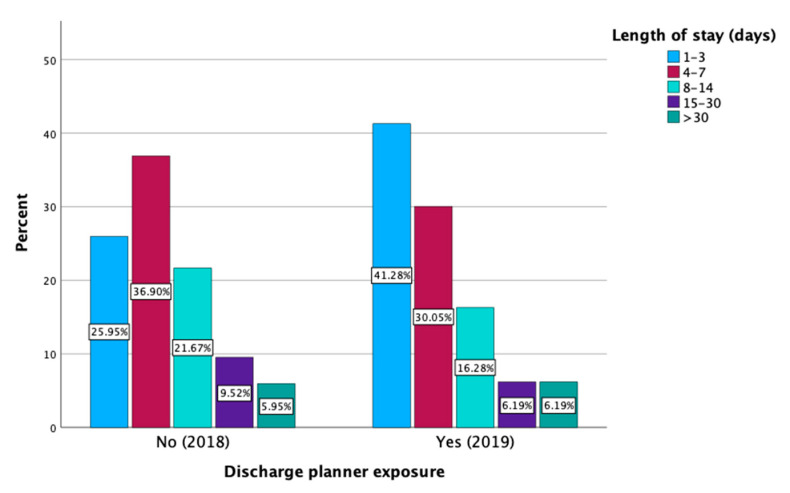
Categorical distribution of length of stay in hospital between 2018 group and 2019 group.

We compared the distribution of the LOS categories between the two groups. The results showed a significant difference in the distribution of the LOS categories between the two groups (*p* < 0.001). The percentage of patients with a LOS of 1–3 days increased from 26% in the 2018 group to 41% in the 2019 group. Conversely, there were decreases in the proportions of patients staying 4–7 days (from 37% to 30%) 8–14 days (from 22% to 16%), and 15–30 days (from 10% to 6%) (Figure 2 and Table S4).

We performed a subgroup analysis to investigate the effect of the discharge planning team on the LOS across different neurological conditions. For ischemic stroke patients, there was a significant reduction in the median LOS from 6 days in the 2018 group to 4 days in the 2019 group (*p* < 0.001). However, after adjustment for confounding factors, the observation became statistically insignificant (β = 1.22, 95% CI [−1.69, 4.13], *p* = 0.411). Patients admitted due to an MS relapse experienced a shorter LOS after adjusting for confounding factors (β = −2.15, 95% CI [−3.79, −0.51], *p* = 0.0125). However, there was no significant difference between the two groups in patients diagnosed with a hemorrhagic stroke, a seizure, a myasthenia gravis crisis, meningitis, and TIA (Table 2).

### 3.3. Readmission Rates

We compared the readmission rates between the two groups. Our analysis demonstrated that 169 (39%) patients were readmitted in the 2019 group compared to 196 (47%) patients readmitted in the 2018 group with an adjusted OR = 0.70, 95% CI [0.49–0.99], *p* = 0.0442. Moreover, we compared the distribution of readmission rate categories between the two groups. The analysis revealed an insignificant difference in the distribution of readmission rate categories between the two groups (*p* = 0.2819). There were decreases in the proportions of patients with readmission within one month after discharge (from 21% in the 2018 group to 18% in the 2019 group) and more than 12 months after discharge (from 21% in the 2018 group to 17% in the 2019 group). Conversely, the rates of readmission increased from 57% in the 2018 group to 65% in the 2019 group within 2–12 months post-discharge (Table 3).

### 3.4. Same-Admission Mortality

In the 2018 group, 20 out of 418 patients (5%) died during the same admission. However, in the 2019 group, 26 out of 436 patients (6%) died during the same admission. The adjusted OR revealed no difference between the two groups (OR = 2.83, 95% CI [0.72, 11.19], *p* = 0.1370) (Table 4).

### 3.5. Overall Mortality

We compared the overall mortality rates between the two groups. In the 2018 group, 57 out of 416 patients (14%) died, while in the 2019 group, 50 out of 436 patients (12%) died. Although there was a slight decrease in the overall mortality rate from 2018 to 2019, there was no difference after adjusting for confounders (OR = 0.99, 95% CI [0.55, 1.82], *p* = 0.9978) (Table 4).

## 4. Discussion

Our study found that the involvement of a discharge planning team was not significantly associated with a reduction in the LOS for patients admitted under the neurology service at our institution after controlling for confounding factors. However, patients who interacted with the discharge planning team were more likely to be discharged within three days after admission. Furthermore, a subgroup analysis indicated that these benefits were particularly pronounced among patients admitted with multiple sclerosis relapse. Furthermore, implementation of the discharge planning team was associated with a 30% lower rate of readmissions compared to those who did not receive such an intervention. Moreover, we found no significant relationship between the involvement of the discharge planning team and either same-admission or overall mortality rates.

The discharge planning team is fundamentally designed to optimize the benefits of hospitalization by utilizing all available resources within the healthcare facility. This team takes on administrative responsibilities, allowing physicians to focus solely on patient care. Moreover, the discharge planning team aims to reduce inefficiencies, such as time loss, by enhancing communication across various departments. By improving communication among healthcare providers, these teams organize the transition from hospital to home, especially for elderly patients. Many reports have demonstrated that effective discharge planning can lead to a reduction in dependency and healthcare costs [19,20,21].

Evidence suggests that the establishment of a discharge planning team can lower readmission rates and enhance patient outcomes, ultimately resulting in considerable cost savings within healthcare settings [22]. Our findings demonstrate that the implementation of a discharge planning team was associated with reduced overall readmission rates. This is in line with previous reports demonstrating that structured discharge planning can effectively reduce readmissions. A systematic review by Braet et al. found that comprehensive discharge planning interventions reduced readmission rates by 22% in various patient populations [13]. Similarly, Jack et al. demonstrated that nurse-led discharge planning protocols resulted in significantly lower readmission rates within 30 days after discharge by approximately 30% among patients admitted to an academic medical center [23]. We observed a trend toward lower early readmission rates (within one month) in the 2019 group, although this difference did not reach statistical significance. This finding suggests that the discharge planning team was effective in ensuring appropriate discharge timing and adequate post-discharge support. The reduction in readmission rates could be attributed to several factors. First, discharge planning teams typically provide better patient education and self-management instructions, which are crucial for preventing complications and unnecessary readmissions [23]. Second, these teams often establish better coordination with community healthcare providers and ensure appropriate follow-up care arrangements [24]. Finally, discharge planners perform comprehensive pre-discharge assessments to identify potential barriers such as mobility limitations, social support needs, or medication management issues, allowing them to implement preventive interventions prior to these challenges precipitating readmissions [25].

The LOS as an outcome that is likely influenced by various confounding variables. Patients with well-controlled or medically stable conditions, who are admitted primarily for neurological evaluation, tend to experience a shorter LOS, as the discharge planning team typically facilitates a more expedited diagnostic workup. Conversely, patients with poorly controlled or complex medical conditions are predisposed to longer stays, even if the neurological assessment is conducted promptly, due to the severity of their comorbidities. This may account for the absence of a significant difference in the overall mean LOS after controlling for potential confounders. Nevertheless, a categorical analysis revealed distinct cohort-level differences. In the 2018 cohort, only 26% of patients experienced a LOS of 1–3 days, whereas this proportion increased significantly to 41% in the 2019 cohort. Simultaneously, the percentage of patients with a LOS of 4–7 days decreased from 36.9% in 2018 to 30% in 2019. These shifts in the distribution of the LOS highlight the potential impact of discharge planning teams in facilitating the timely discharge of patients once their evaluation was completed, particularly for those with favorable medical conditions. These results align with findings from Fjaertoft et al., which indicated that patients receiving coordinated discharge planning had shorter hospitalizations compared to those without such support [26]. Furthermore, research by Katikireddi et al. similarly demonstrated that multidisciplinary discharge planning not only shortened the LOS but also reduced hospital costs and enhanced overall patient satisfaction [27]. Additionally, a systematic review by Shepperd and colleagues found that patients who benefited from structured discharge planning experienced shorter hospital stays and a lower risk of readmission [17]. These findings emphasize the vital role of discharge planning teams in reducing hospitalization durations, minimizing complications associated with hospital stays, optimizing resource utilization, and improving patient satisfaction by facilitating a more efficient transition back to the home environment.

Our multiple regression analysis revealed that ICU admission was significantly associated with an increased LOS. This aligns with a previous study finding that ICU admissions substantially extend hospital stays due to an increased illness severity and complications [28]. Regarding comorbidities, liver cirrhosis showed a significant impact on the LOS, with a notable interaction effect with discharge planning implementation. This interaction suggests that patients with liver cirrhosis may benefit less from discharge planning interventions, consistent with previous findings that complex comorbidities create additional barriers to effective discharge planning [3,10]. Hospital-acquired complications, particularly dysphagia and infections requiring IV antibiotics, significantly extended the LOS. The interaction analysis revealed that the effectiveness of discharge planning varied based on IV antibiotic requirements. This aligns with Gonçalves-Bradley et al.’s systematic review showing that discharge planning’s impact diminishes with increasing medical complexity [7]. These secondary findings provide a crucial context for interpreting the primary outcomes. The reduced effectiveness of discharge planning in patients with specific comorbidities and complications explains why the overall LOS showed no significant difference despite improvements in early discharge rates (1–3 days).

This study demonstrates that the implementation of a discharge planning team significantly reduces the LOS for patients admitted with multiple sclerosis relapse after adjusting for confounders. However, this effect was not clinically significant for other conditions, including ischemic strokes, hemorrhagic strokes, seizures, myasthenia gravis crises, meningitis, and transient ischemic attacks. These findings indicate that the effectiveness of discharge planning teams can vary depending on the specific medical conditions, the complexity involved in their management, and the types of interventions required during hospitalization. The effectiveness of discharge planning in MS relapse cases likely stems from the relatively standardized treatment protocols, which typically center around steroid administration and systematic monitoring. MS relapses often follow more predictable clinical trajectories, allowing for more structured discharge planning processes. Importantly, this finding should be interpreted cautiously since it is based on a small sample size (53 patients). The loss of statistical significance in the LOS reduction for ischemic stroke patients after adjustment for confounding factors reflects the complex nature of stroke care. As demonstrated in the Cochrane review by Langhorne et al., stroke care frequently involves managing multiple concurrent issues beyond the primary neurological condition [11]. Nonetheless, discharge planning remains a critical aspect of patient care, ensuring continuity and optimizing post-discharge outcomes across all diagnoses. These results emphasize the need for customized discharge planning strategies that address the unique needs and recovery pathways of different patient populations.

Our findings indicate that there is no definitive association between the involvement of a discharge planning team and mortality rates. The lack of significant mortality differences aligns with previous systematic reviews examining discharge planning interventions. A Cochrane review by Gonçalves-Bradley et al. found no clear evidence that discharge planning impacts mortality rates (risk ratio = 1.02, 95% CI [0.83 to 1.27], *p* = 0.82) [7]. Similarly, Shepperd et al. observed that the effects of implementing discharge planning on mortality rates remain unclear [17]. The lack of mortality impact should not be interpreted as a limitation of discharge planning effectiveness, as the primary goals of discharge planning are to improve care transitions, reduce the length of stay, and prevent readmissions rather than directly impact survival. Further investigations with longer study durations are warranted to explore the effects on mortality.

The implementation of a discharge planning team not only reduces the length of stay but also significantly enhances patients’ quality of life, particularly regarding their mental and emotional well-being. A study by Rich et al. found that patients who received structured discharge planning reported better mental health outcomes [29]. Furthermore, research conducted by Preen et al. indicated that a multidisciplinary discharge care plan, initiated prior to discharge, improves quality of life, patient engagement, and satisfaction with the discharge process. These findings suggest that the benefits of discharge planning extend beyond the immediate reduction in hospital stay, significantly contributing to overall patient satisfaction and recovery [30].

The impact of health systems, both within and surrounding hospitals, on patient outcomes is significant and multifaceted. As highlighted by Baskar et al., systems-based approaches have been shown to improve in-hospital temporal parameters and maximize the utility of acute reperfusion therapies in acute ischemic stroke. Also, they demonstrate that identifying gaps in acute workflow, variations in processes, and challenges in implementation is essential for systems-based interventions to be effective [31]. Moreover, integrated care systems that extend beyond hospital buildings have been shown to improve patient outcomes, reduce readmissions, and enhance overall quality of care [32]. The implementation of transitional care models, which bridge the gap between hospital and home care, has demonstrated a reduction in 30-day readmission rates [33]. Additionally, the adoption of electronic health records and health information exchanges across health systems has been associated with improved care coordination and reduced medical errors [34]. These system-level interventions highlight the importance of considering the entire healthcare system when evaluating patient outcomes, rather than focusing only on in-hospital care. Future studies are warranted to investigate how different health system structures and integration levels influence outcomes for patients with neurological conditions.

Some limitations of this study warrant consideration. First, while we acknowledged the single-center design, this limitation extends further as our institution’s unique characteristics as a tertiary care center and our discharge planning team’s specific organizational structure may limit the generalizability of our findings to other healthcare settings. Second, a non-random convenience sampling technique was employed, which might introduce selection bias. However, we included all patients admitted and discharged under the neurology service from January 2018 to December 2019, rather than selecting specific cases. Third, various factors influencing the LOS extend beyond the implementation of a discharge planning team. Notably, the necessity for ICU admission significantly affects the hospitalization duration, as patients requiring intensive care often face more severe health challenges and longer recovery times. Furthermore, comorbid conditions, such as MI, can lead to extended hospital stays due to the complexity and intensity of the treatments required. To address these confounding factors, we employed a multiple regression analysis, which indicated no overall difference in the LOS. Fourth, we did not account for confounding external factors in the multiple linear regression analysis such as changes in hospital policies and protocols, and variations in staffing patterns. Important patient-level variables such as socioeconomic status, family support systems, and health literacy levels were not captured in our dataset, potentially affecting both the length of stay and readmission rates. Thus, further studies are warranted to consider these external factors in the analysis. Lastly, the limited number of patients with a hemorrhagic stroke, multiple sclerosis, myasthenia gravis, seizures, meningitis, and a transient ischemic attack restricted our conclusive findings in these subgroups. Therefore, future multi-center studies with larger sample sizes for specific neurological conditions are needed to better evaluate the effectiveness of discharge planning across different neurological diseases.

## 5. Conclusions

The findings of our study demonstrate that implementing a discharge planning system in neurological services yielded several important benefits. Patients in the 2019 group were significantly more likely to achieve early discharge within three days compared to the 2018 group, with this effect being particularly pronounced in patients with multiple sclerosis relapse. Importantly, exposure to the discharge planning system was associated with lower readmission rates, suggesting improved care transitions and post-discharge outcomes. The observed improvements in early discharge rates and reduced readmissions, achieved without compromising patient outcomes (mortality), suggest that systematic discharge planning can effectively enhance healthcare delivery efficiency while potentially reducing healthcare costs and improving bed availability for emergency admissions.

## Figures and Tables

**Table 1:** Baseline characteristics of the included patients.

Variable	2018 = 420	2019 = 436	*p*-Value
Age, median (IQR)	63 (47–75)	62 (46–74)	0.5512
Gender, n (%)			
Male	251 (60%)	234 (54%)	0.0845
Female	169 (40%)	202 (46%)	
Body mass index, n (%)			
Underweight	22 (5%)	25 (6%)	0.3423
Normal	120 (29%)	121 (28%)	
Overweight	132 (32%)	133 (31%)	
Obese	102 (24%)	90 (21%)	
Extremely obese	43 (10%)	64 (15%)	
Diabetes, n (%)	212 (51%)	211 (48%)	0.5844
Hypertension, n (%)	235 (56%)	239 (55%)	0.7833
Dyslipidemia, n (%)	70 (17%)	90 (21%)	0.1374
Stroke, n (%)	92 (22%)	95 (22%)	0.9673
Transient ischemic attack, n (%)	7 (2%)	8 (2%)	0.8512
Atrial fibrillation, n (%)	20 (5%)	27 (6%)	0.3723
Heart failure, n (%)	21 (5%)	23 (5%)	0.8781
Chronic kidney disease, n (%)	21 (5%)	32 (7%)	0.1596
Coronary artery disease, n (%)	41 (10%)	57 (13%)	0.134
Liver cirrhosis, n (%)	2 (1%)	4 (1%)	0.6867
Hemodialysis, n (%)	2 (1%)	5 (1%)	0.4518
Peripheral vascular disease, n (%)	5 (1%)	4 (1%)	0.7484
Barthel index, median (IQR)	12 (5–19)	12 (4–18)	0.8602

**Table 2:** Subgroup analysis of length of stay in hospital based on neurological diseases.

Diagnosis	Discharge Planner Exposure	Median	IQR	*p*-Value	Adjusted β Coefficient (95% CI) *	*p*-Value
Ischemic Stroke	No	6	4–10	<0.001	1.22 (−1.69, 4.13)	0.411
	Yes	4	3–7			
Hemorrhagic stroke	No	6	3–18	0.7788	−0.96 (−14.71, 12.79)	0.8825
	Yes	7	2–12			
TIA	No	3	2–4	0.3525	0.53 (−0.54, 1.66)	0.3051
	Yes	3	2–8			
Seizure	No	4	3–6	0.8249	−4.64 (−18.45, 9.16)	0.4993
	Yes	4	2–10			
Multiple sclerosis relapse	No	5	3–13	0.0739	−2.15 (−3.79, −0.51)	0.0125
	Yes	3	2–7			
Myasthenia gravis crisis **	No	10	7–14	0.5731	3.51 (−126.09, 133.12)	0.7888
	Yes	10	4–15			
Meningitis	No	7	3–15	0.6044	2.89 (−1.22, 7)	0.1406
	Yes	6	3–13			

IQR, interquartile range; CI, confidence interval; TIA, transient ischemic attack. * Adjusted model variables include age, gender, BMI, diabetes, hypertension, dyslipidemia, stroke, transient ischemic attack, atrial fibrillation, heart failure, chronic kidney disease, coronary artery disease, liver cirrhosis, hemodialysis, peripheral vascular disease, immediate ICU admission, ICU admission, pneumonia, urinary tract infection, myocardial infarction, deep vein thrombosis, pulmonary embolism, dysphagia, IV antibiotic requirement, Barthel index. ** The adjusted model variables of myasthenia gravis crisis include age, gender, BMI, diabetes, hypertension, immediate ICU admission, ICU admission, dysphagia, IV antibiotic requirement, Barthel index. The other variables were not included in the model since all the patients were free of them.

**Table 3:** The difference in readmission rates across the periods between 2018 group and 2019 group.

Outcome	2018 Group	2019 Group	Adjusted OR (95% CI) *	*p*-Value
Overall readmission rate, n (%)	196 (47%)	169 (39%)	0.70 (0.49, 0.99)	0.0193
Readmission by months after discharge				
1 month	43 (21%)	30 (18%)	-	0.2819
2–12 months	111 (57%)	110 (65%)	-	
>12 months	41 (21%)	29 (17%)	-	

OR, odds ratio; CI, confidence interval. * Adjusted model variables include age, gender, BMI, diabetes, hypertension, dyslipidemia, stroke, transient ischemic attack, atrial fibrillation, heart failure, chronic kidney disease, coronary artery disease, liver cirrhosis, hemodialysis, peripheral vascular disease, immediate ICU admission, ICU admission, pneumonia, urinary tract infection, myocardial infarction, deep vein thrombosis, pulmonary embolism, dysphagia, IV antibiotic requirement, Barthel index.

**Table 4:** Mortality during the same admission and overall mortality between 2018 group and 2019 group.

Outcome	2018 Group	2019 Group	Adjusted OR (95% CI) *	*p*-Value
Mortality during the same admission	20 (5%)	26 (6%)	2.83 (0.72, 11.19)	0.1370
Overall mortality	57 (14%)	50 (12%)	0.99 (0.55, 1.82)	0.9978

OR, odds ratio; CI, confidence interval. * Adjusted model variables include age, gender, BMI, diabetes, hypertension, dyslipidemia, stroke, transient ischemic attack, atrial fibrillation, heart failure, chronic kidney disease, coronary artery disease, liver cirrhosis, hemodialysis, peripheral vascular disease, immediate ICU admission, ICU admission, pneumonia, urinary tract infection, myocardial infarction, deep vein thrombosis, pulmonary embolism, dysphagia, IV antibiotic requirement, Barthel index.

## Data Availability

Data are available upon reasonable request from the corresponding author.

## Supplementary Materials

The following supporting information can be downloaded at: https://www.mdpi.com/article/10.3390/healthcare13020143/s1, Table S1. Hospital course during admission between 2018 group and 2019 group. Table S2. Multiple linear regression analysis results. Table S3. Interaction analysis results. Table S4. Categorical distribution of length of stay in hospital between 2018 group and 2019 group.
