# Supplementary material for: The Role of the Discharge Planning Team on the Length of Hospital Stay and Readmission in Patients with Neurological Conditions: A Single-Center Retrospective Study
Source: Healthcare (Basel). 2025 Jan 14;13(2):143. doi: 10.3390/healthcare13020143 (PMC11764536; doi:10.3390/healthcare13020143)
Supplement: Supplementary file 1 [file healthcare-13-00143-s001.zip › healthcare-3355303-supplementary.pdf]

Supplementary Data

Table S1. Hospital course during admission between 2018 group and 2019 group.

Variable	2018 = 420	2019 = 436	*P* value
**ICU admission, n (%)**	52 (12%)	20 (5%)	< 0.0001
**Immediate ICU admission, n (%)**	33 (8%)	23 (5%)	0.131
**Pneumonia, n (%)**	40 (10%)	27 (6%)	0.0751
**Urinary tract infection, n (%)**	46 (11%)	23 (5%)	0.0025
**Myocardial infarction, n (%)**	2 (1%)	1 (0.2%)	0.6177
**Deep vein thrombosis, n (%)**	7 (2%)	0 (0%)	0.0067
**Pulmonary embolism, n (%)**	3 (1%)	3 (1%)	0.9633
**Dysphagia, n (%)**	35 (8%)	36 (8%)	0.9677
**Requires intravenous antibiotics, n (%)**	73 (17%)	81 (19%)	0.6574

Table S2. Multiple linear regression analysis results.

	β coefficient	*P* value	Lower 95%	Upper 95%
Discharge planner exposure	-0.584923	0.6007	-2.778316	1.6084696
Age (years)	4.8849826	0.1451	-1.689936	11.459901
Gender, female	1.6999801	0.1369	-0.541323	3.9412827
**BMI**				
Underweight	8.8353818	**0.0247**	1.1269384	16.543825
Normal	-5.36582	**0.01**	-9.441873	-1.289767
Overweight	-0.295537	0.8825	-4.221987	3.6309137
Obese	-0.809796	0.72	-5.244884	3.6252918
Extremely obese	-2.364229	0.3785	-7.632442	2.9039847
**Past medical history**				
Diabetes	-0.124492	0.9242	-2.693356	2.4443728
Hypertension	-1.898553	0.196	-4.77865	0.9815446
Dyslipidemia	-0.136635	0.9258	-3.017148	2.7438782
Stroke	1.0694066	0.4417	-1.658668	3.7974809
Transient ischemic attack	1.3559452	0.7366	-6.558559	9.2704495
Atrial fibrillation	-2.076764	0.4037	-6.957798	2.8042705
Heart failure	-2.119298	0.4401	-7.507237	3.2686408
Chronic kidney disease	-2.904169	0.2522	-7.880563	2.0722258
Coronary artery disease	-1.520897	0.4094	-5.138875	2.0970823
Liver cirrhosis	12.935515	**0.0386**	0.6828548	25.188175
Hemodialysis	-4.091223	0.5803	-18.61322	10.430777
Peripheral vascular disease	-2.779208	0.6193	-13.75826	8.1998418
**Hospital course**				
Immediate ICU admission	-6.443621	**0.0124**	-11.48668	-1.400557
ICU Admission	13.110526	**<.0001**	8.1461959	18.074856
Pneumonia	1.436818	0.583	-3.700037	6.5736729
Urinary tract infection	0.3359811	0.8763	-3.902255	4.5742166
Myocardial infarction	5.6316795	0.6839	-21.52265	32.786007
Deep vein thrombosis	10.836882	0.087	-1.577191	23.250955
Pulmonary embolism	0.5216227	0.9271	-10.67719	11.720433
Dysphagia	4.7237161	**0.0242**	0.6184814	8.8289508
Required IV antibiotics	9.4616658	**<.0001**	5.9485577	12.974774
Barthel index	-16.90156	**0.0045**	-28.54564	-5.257488

Table S3. Interaction analysis results.

	**β coefficient**	***P* value**	**Lower 95%**	**Upper 95%**
**Group*Liver cirrhosis**	14.387402	0.0243	1.8732566	26.901548
**Group*Immediate ICU admission**	5.606053	0.0335	0.4383606	10.773745
**Group*ICU Admission**	-3.939816	0.1572	-9.402367	1.5227364
**Group*Dysphagia**	-2.47676	0.2234	-6.467666	1.5141462
**Group*Required IV antibiotic**	-3.350119	0.0363	-6.485647	-0.214591
**Group*Barthel index**	-0.015984	0.9098	-0.292864	0.2608967

Table S4. Categorical distribution of length of stay in hospital between 2018 group and 2019 group.

LOS (days)	Discharge planner exposure				*P* value
	No (2018)		Yes (2019)		
	N	N%	N	N%	
**1-3**	109	26%	180	41%	<0.001
**4-7**	155	37%	131	30%	
**8-14**	91	22%	71	16%	
**15-30**	40	10%	27	6%	
**>30**	25	6%	27	6%	
